# Uncovering the lignin-degrading potential of *Serratia quinivorans* AORB19: insights from genomic analyses and alkaline lignin degradation

**DOI:** 10.1186/s12866-024-03331-3

**Published:** 2024-05-25

**Authors:** Nadia Sufdar Ali, Subarna Thakur, Mengwei Ye, Fanny Monteil-Rivera, Youlian Pan, Wensheng Qin, Trent Chunzhong Yang

**Affiliations:** 1 Aquatic and Crop Resource Development Research Centre, National Research Council Canada, Ottawa, ON Canada; 2 Department of Biology, Lakehead University, Thunder Bay, ON Canada; 3 Department of Bioinformatics, University of North Bengal, Siliguri, India; 4 Aquatic and Crop Resource Development Research Centre, National Research Council Canada, Montreal, QC Canada; 5 Digital Technologies Research Centre, National Research Council Canada, Ottawa, ON Canada; 6 BioWise Technologies Inc, Ottawa, Canada

**Keywords:** Lignin degradation, CAZymes, *Serratia quinivorans* AORB19, LC–UV, Whole genome analyses, Lignocellulosic waste

## Abstract

**Background:**

Lignin is an intricate phenolic polymer found in plant cell walls that has tremendous potential for being converted into value-added products with the possibility of significantly increasing the economics of bio-refineries. Although lignin in nature is bio-degradable, its biocatalytic conversion is challenging due to its stable complex structure and recalcitrance. In this context, an understanding of strain's genomics, enzymes, and degradation pathways can provide a solution for breaking down lignin to unlock the full potential of lignin as a dominant valuable bioresource. A gammaproteobacterial strain AORB19 has been isolated previously from decomposed wood based on its high laccase production. This work then focused on the detailed genomic and functional characterization of this strain based on whole genome sequencing, the identification of lignin degradation products, and the strain’s laccase production capabilities on various agro-industrial residues.

**Results:**

Lignin degrading bacterial strain AORB19 was identified as *Serratia quinivorans* based on whole genome sequencing and core genome phylogeny. The strain comprised a total of 123 annotated CAZyme genes, including ten cellulases, four hemicellulases, five predicted carbohydrate esterase genes, and eight lignin-degrading enzyme genes. Strain AORB19 was also found to possess genes associated with metabolic pathways such as the β-ketoadipate, gentisate, anthranilate, homogentisic, and phenylacetate CoA pathways. LC–UV analysis demonstrated the presence of p-hydroxybenzaldehyde and vanillin in the culture media which constitutes potent biosignatures indicating the strain’s capability to degrade lignin. Finally, the study evaluated the laccase production of *Serratia* AORB19 grown with various industrial raw materials, with the highest activity detected on flax seed meal (257.71 U/L), followed by pea hull (230.11 U/L), canola meal (209.56 U/L), okara (187.67 U/L), and barley malt sprouts (169.27 U/L).

**Conclusions:**

The whole genome analysis of *Serratia quinivorans* AORB19*,* elucidated a repertoire of genes, pathways and enzymes vital for lignin degradation that widens the understanding of ligninolytic metabolism among bacterial lignin degraders. The LC-UV analysis of the lignin degradation products coupled with the ability of *S. quinivorans* AORB19 to produce laccase on diverse agro-industrial residues underscores its versatility and its potential to contribute to the economic viability of bio-refineries.

**Supplementary Information:**

The online version contains supplementary material available at 10.1186/s12866-024-03331-3.

## Background

Next to cellulose, lignin is the most generic renewable material available on earth, and the primary by-product of lignocellulosic bio-refineries such as pulp and paper, bioethanol and biogas industries. It is a major component found in plant cell walls in association with cellulose and hemicellulose and act as a physical and chemical barrier to biodegradative systems [1]. Lignin is a crosslinked aromatic polymer comprised of various hydroxy phenylpropanoid units, namely syringyl (S), guaiacyl (G) or *p*-hydroxyphenyl (H) propanoid units, linked together through ether and carbon–carbon bonds [2]. Due to its high relative abundance and its high content of phenolic units, lignin is seen as the substrate of choice for the production of value-added aromatic biochemicals.

Microbial and enzymatic lignin degradations hold great potential for the development of bioprocesses which offers sustainable and selective alternatives to conventional thermochemical production [3–5]. However, despite this theoretical potential, lignin valorization into aromatic monomers via thermochemical or biological processes remains limited [2, 6, 7]. One of the major challenges in biological lignin conversion is the complexity of lignin and the fact that proficient lignin degradation requires a range of multiple enzymes working in cascade reactions. So far, enzymes that degrade monomeric phenolic units have been reported to degrade/modify lignin with only low efficiency. Identifying microbial catalysts with enhanced lignin-degrading capabilities is crucial for breaking down lignin into usable fragments and achieving high productivity in bioconversion of lignin.

*Serratia* is a gram-negative bacterial genus belonging to the large and diverse *Yersiniaceae* family. There are currently more than 20 recognized species within the genus *Serratia*, which are differentiated by their characteristics, including morphology, physiology, and biochemical properties. Due to its relevance as an opportunistic human pathogen, much of the research on this genus has focused on the *Serratia marcescens*. Other related species including *S. rubidaea, S. odorifera*, and *S. liquefaciens* have been also reported to be associated with nosocomial infections. *Serratia quinivorans* was previously classified as a subspecies of *S. proteamaculans* and designated as *S. proteamaculans subsp. quinivorans* [8]. It was recently included as a separate species in *Serratia liquefaciens* complex, which contains other species like *S. liquefaciens, S. grimesii* and *S. proteamaculans* [9], based on average nucleotide identity (ANI) phylogroup analysis. The sub-specific epithet "quinovora" in *S. proteamaculans subsp. quinivorans* means "quinine-devouring," indicating the strain’s ability to utilize quinate [10]. They are gram negative, non-pigmented, non-spore-forming rods, facultative anaerobic, motile by means of peritrichous flagella and have been mostly isolated from soil, plants, wild rodents, insects, and water [9].

Recent studies highlight *Serratia quinivorans* as a versatile microorganism with potential applications in diverse fields. Previous studies have reported compelling evidence of the remarkable cold-adaptation and biocontrol capabilities of *Serratia quinivorans* PKL:12 [11]. In addition, the discovery of a psychrotolerant strain of *S. quinivorans*, which secretes β-D-galactosidase that remains active in cold environments, highlights the potential of this species for future biotechnological applications [12]. Furthermore, a novel endophytic strain of *S. quinivorans*, KP32 was identified which has the ability to produce a variety of lytic and antioxidant enzymes, highlighting its immense potential as a biocontrol agent with promising applications in several fields [13]. Notably, a *S. quinivorans strain* 124R was found associated with aromatic metabolism while using organosolv lignin as the sole carbon source under anoxic conditions, demonstrating the potential of *S. quinivorans* strains for lignin biorefinery applications [14].Whole genome sequencing can provide insights into the metabolic pathways and regulatory mechanisms involved in the process and is important for advancing the understanding of lignin degradation and its potential applications in the production of biochemicals. For example, in a recent study of the lignin-degrading bacteria *Streptomyces thermocarboxydus* DF3-3, sequencing of the whole genome allowed for the identification of key regulatory genes and pathways involved in the degradation of lignin [15]. Strain *Serratia* sp*.* (AORB19) was recently isolated by our lab from decomposed wood samples that is able to grow on lignin-based medium and produce laccase enzyme [16].

Industrial processes generate significant lignocellulosic wastes, including agro-industrial residues, palm oil mill effluents, brewing industry by-products, and dairy industry residues [17, 18]. Consequently, improper disposal or incineration of these wastes exacerbates soil and water quality deterioration and contributes to air pollution [19, 20]. In this backdrop, employing microbial strains capable of utilizing these wastes not only mitigates environmental challenges associated with waste management but also has the potential to yield value-added products and biologically active compounds [21–24]. In line with this aim, recent studies have reported the efficacy of microbial strains in the laccase production utilizing various agro-industrial residues [25–27]. However, there is a limited understanding of the strain *Serratia quinivorans* in terms of its underlying molecular mechanisms of lignin degradation, and laccase production capabilities in diverse agro-industrial residues in waste valorization applications, particularly in lignocellulosic biorefineries. Therefore, this study aims to investigate the lignin-degrading capabilities of strain AORB19 employing whole-genome sequencing and analysis of its degradative products. Additionally, it will assess the effectiveness of strain AORB19 in enhancing laccase production using various agro-industrial residues.

## Results and discussion

### General genomic features of *Serratia* AORB19

The final *Serratia* AORB19 genome assembly contained 42 contigs sequences longer than 500 bp with a total consensus genome size of 5587018 bp (∼5.5 Mb) and GC content of 54.99%, which was comparable to the genome sizes of previously sequenced *Serratia* genomes. The largest contig size was 1236132 bp, and the N50 size was 370852 bp. The BUSCO evaluation showed 99.7% completeness of this genome, indicating a high-quality genome assembly. All annotation statistics are listed in Table 1.

**Table 1:** General features of strain AORB19 genome

Features	
Length in bp	5587018
GC content (%)	54.99
Number of Contigs (> 500 bp)	42
N50 Values (bp)	370852
N90 Values (bp)	86520
L50 Values (bp)	4
L90 Values (bp)	13
Largest contig length (bp)	1236132
Number of protein coding genes	5655
Number of tRNAs	99
Number of protein-encoding genes with functional assignment	4002
Number of protein-encoding genes without functional assignment	1653
% Features that are in Local Protein Families	86.37
% Features that are Hypothetical	29.23

**Fig. 1:**
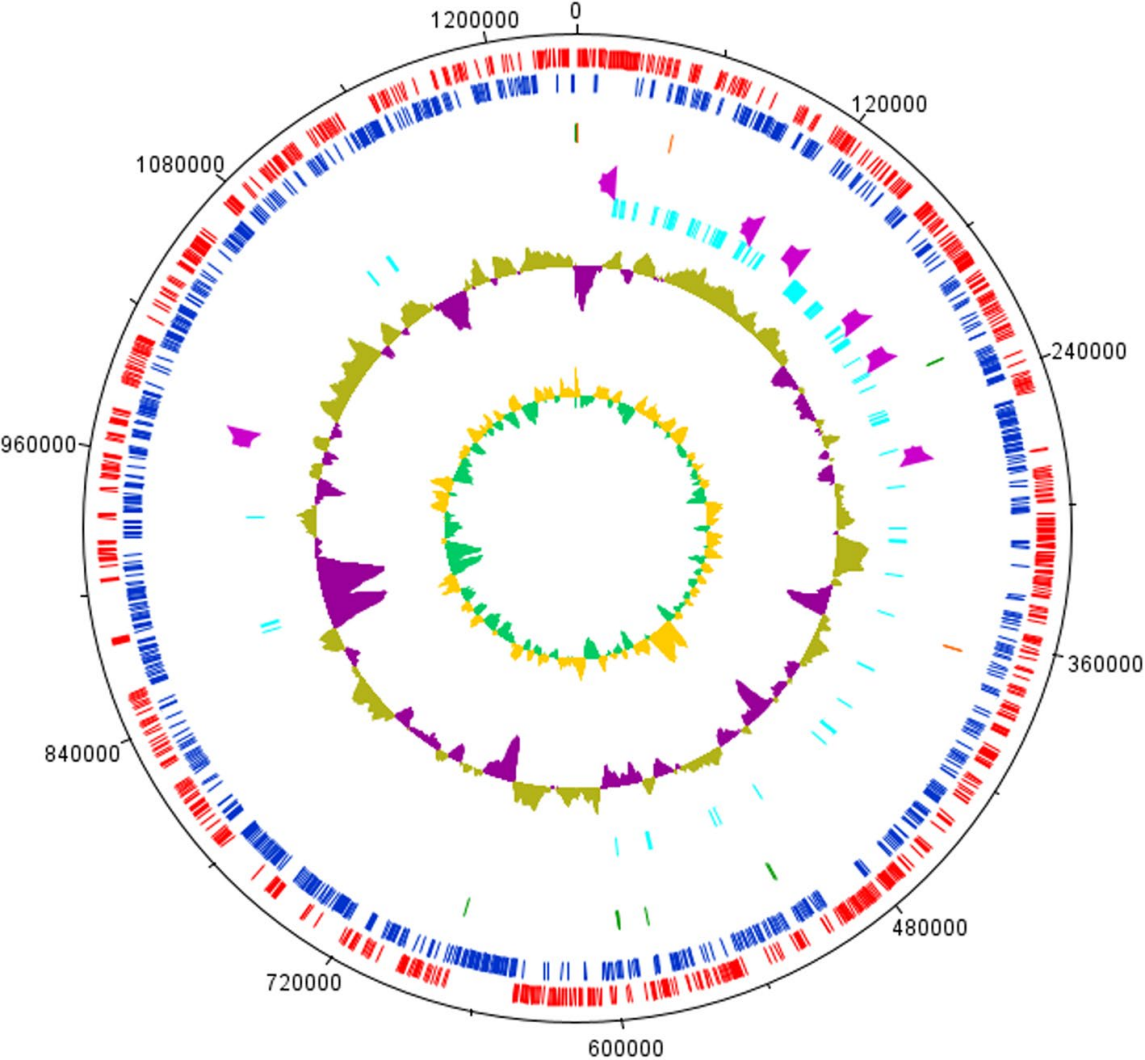
Overview of *S. quinivorans* AORB19 genome. **A**: Circular plot generated by DNA plotter. Circles indicate, from inside outwards: GC skew (yellow/green); GC content (moss green/purple); putative lignocelluloses enzymes (cyan); putative laccase –like enzyme genes (purple); tRNA coding genes (green) and rRNA (red); protein-coding genes on reverse strand (blue); and protein-coding genes on forward strand (red)

The genome had 5655 protein-coding genes, of which 4002 genes were functionally assigned, while the remaining genes were annotated as hypothetical proteins (29.23%). In addition, 108 non-coding RNAs, including tRNAs, rRNAs, and snRNA were identified in the *Serratia* genome. The circular plot of the genome is represented in Fig. 1.

**Fig. 2:**
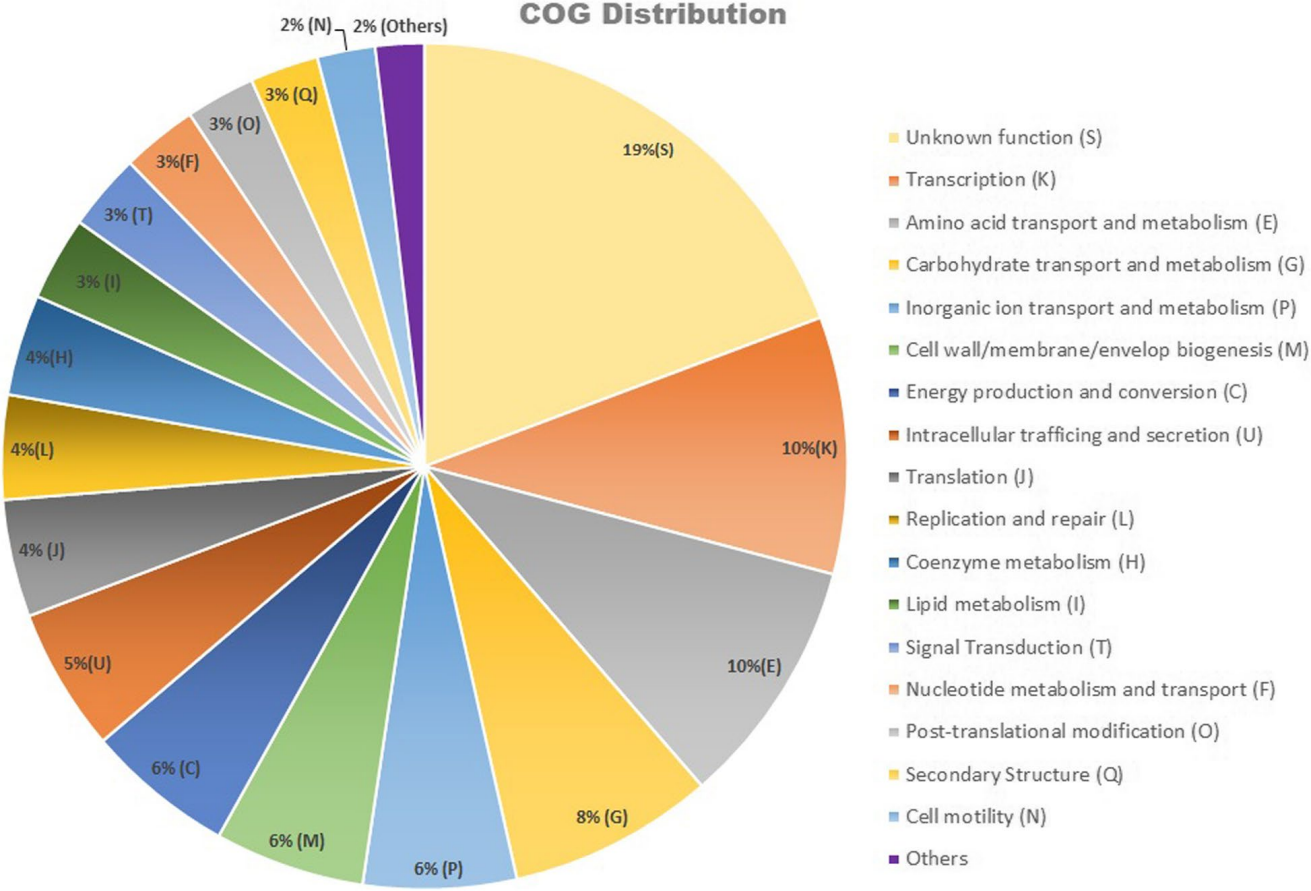
Distribution of Clusters of Orthologous Genes (COG) functional categories in the complete genome sequence of *S quinivorans* AORB19 genome

**Fig. 3:**
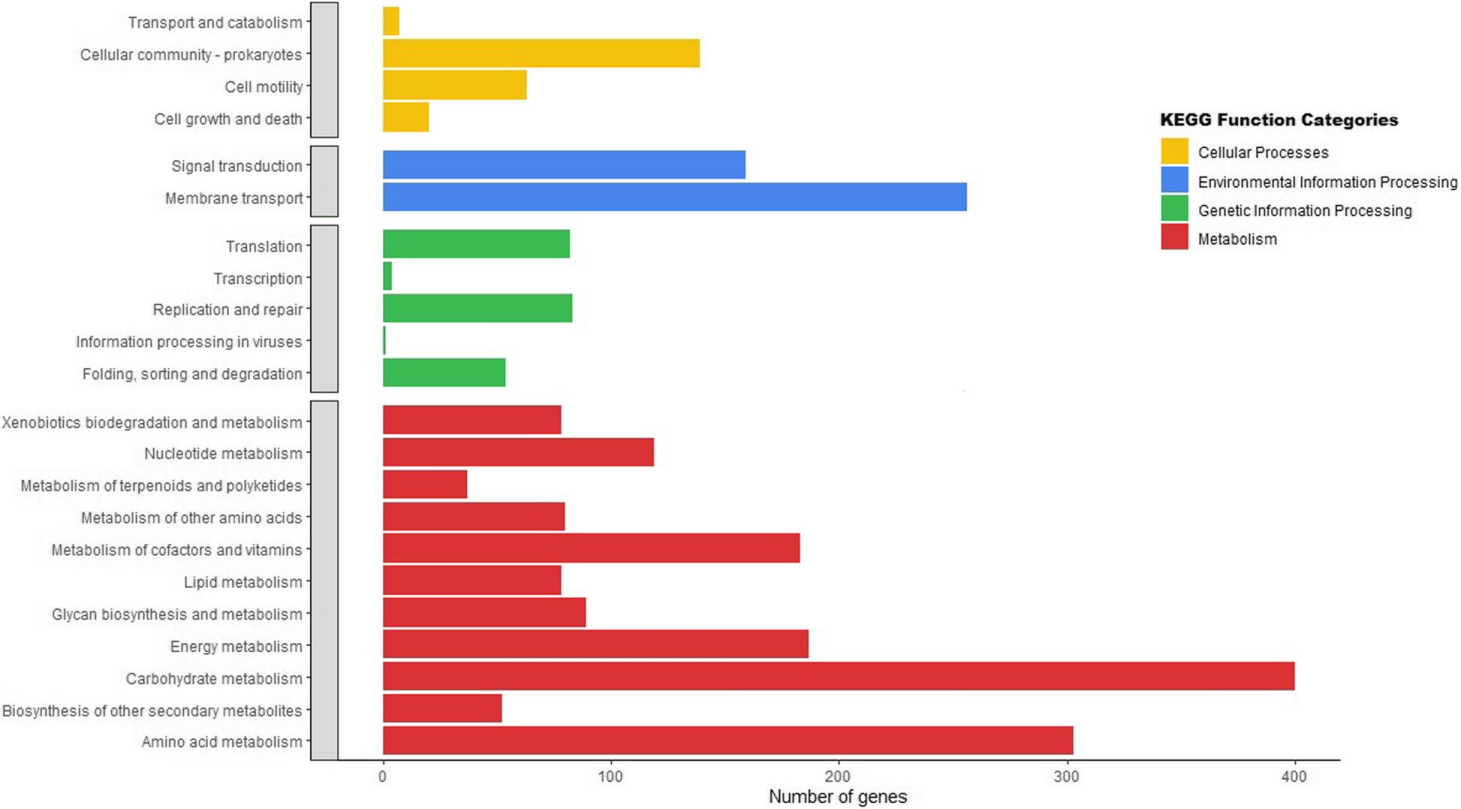
Kyoto Encyclopedia of Genes and Genome (KEGG) pathway annotation of the assembled genome of strain AORB19. Percentage of gene sequences assigned to each sub-category of the four top KO categories, namely metabolism (red), genetic information processing (green), environmental information processing (blue), cellular processes (yellow) were calculated and displayed

**Fig. 4:**
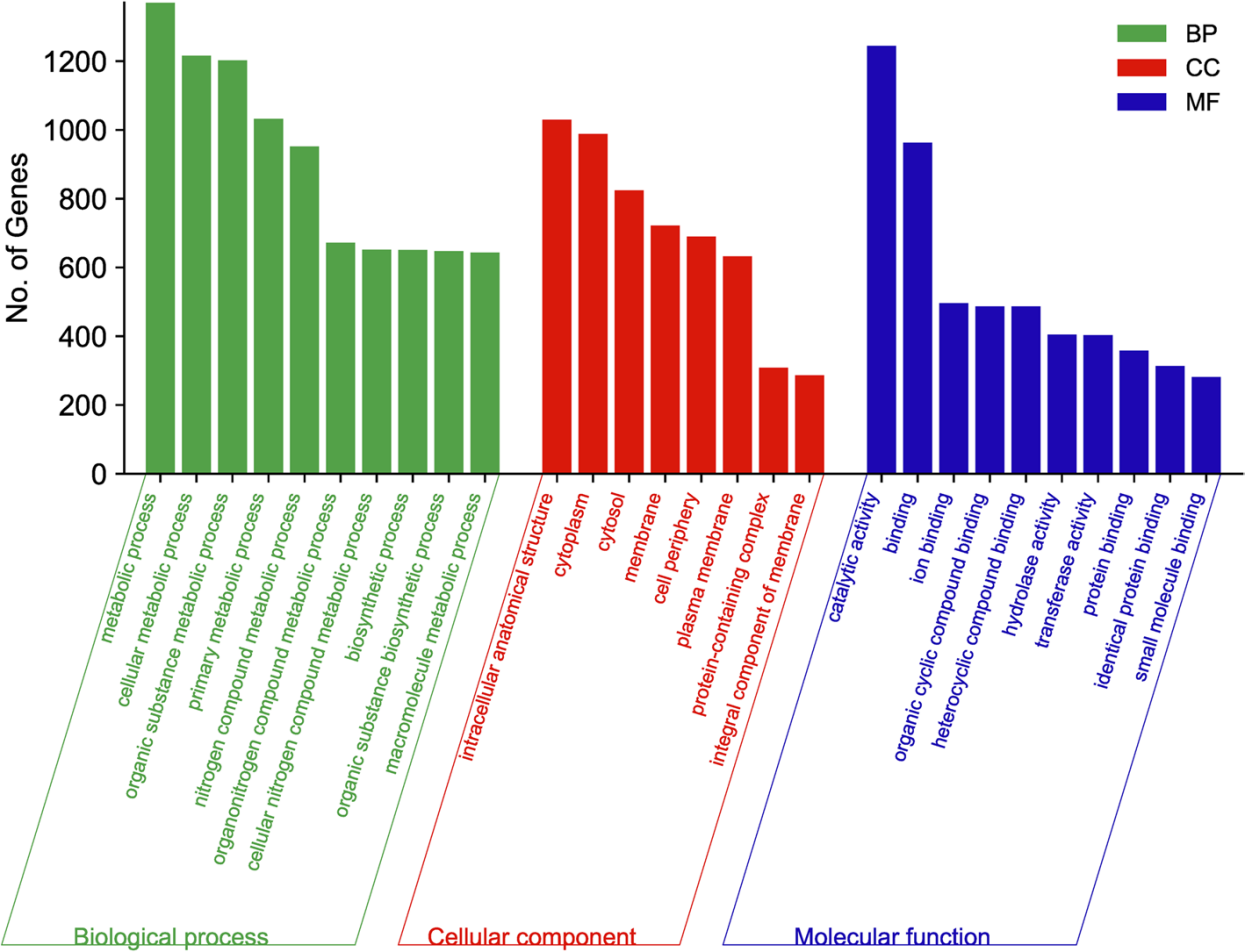
GO classification of bacterial gene function annotation. MF stands for molecular function; BP for biological process and CC for cellular components

The functional annotation reveals that out of the total protein-coding genes, 4955 genes that have been assigned were annotated with Clusters of Orthologous Genes (COG) terms and grouped into 21 classes, generally covering the essential functions of cellular processes, metabolism, information storage and general function. The top five functional COG terms were transcription, K (10%), amino acid transport and metabolism, E (10%), carbohydrate transport and metabolism, G (8%), inorganic ion transport and metabolism, P (6%), and cell wall/membrane/envelope biogenesis, M (6%) (Fig. 2). In the Kyoto Encyclopedia of Genes and Genomes (KEGG) annotation of the genome, 3320 genes were successfully annotated with 2333 KEGG Orthologous (KO) terms. Detailed analysis of the KEGG pathways (Fig. 3) revealed that the genes were enriched in 23 pathways and four major functions of metabolism, including genetic information processing, environmental information processing and cell process. In the category of metabolic processes, 11 pathways were annotated among which 400 and 303 genes were enriched in the carbohydrate metabolism and amino acid metabolism pathways, respectively. Five pathways in the genetic information processing category were annotated, with the translation pathway having the most annotations of 82 genes. Under the environmental information processing category, 256 and 159 genes were enriched in the membrane transport and signal transduction pathways, respectively. A total of 2366 genes from AORB19 were annotated in the Gene ontology (GO) database under the three broad categories of biological process (BP), cellular component (CC), and molecular function (MF). As shown in Fig. 4, the top three annotated BPs were the metabolic processes, cellular metabolic process and organic substance metabolic process, in which 1370, 1216, and 1202 genes were enriched, respectively. There were 1030, 988, and 824 genes enriched in the top three CCs, including intracellular anatomical structure, cytoplasm and cytosol respectively. The top annotated MFs include catalytic activity, binding and ion-binding with 1244, 963 and 496 genes.

### Whole genome comparison

The GenBank now contains numerous whole-genome sequences due to advancements in next-generation sequencing, enabling the utilization of whole-genome sequencing as a novel approach for species differentiation. Average Nucleotide Identity (ANI) and *in-silico* DNA-DNA hybridization (*is*DDH) analysis are the most commonly used parameters for taxonomic assignments. In the context of genome-based species delineation, *in-silico* DDH is widely regarded as a reliable alternative to traditional DDH methods.

**Fig. 5:**
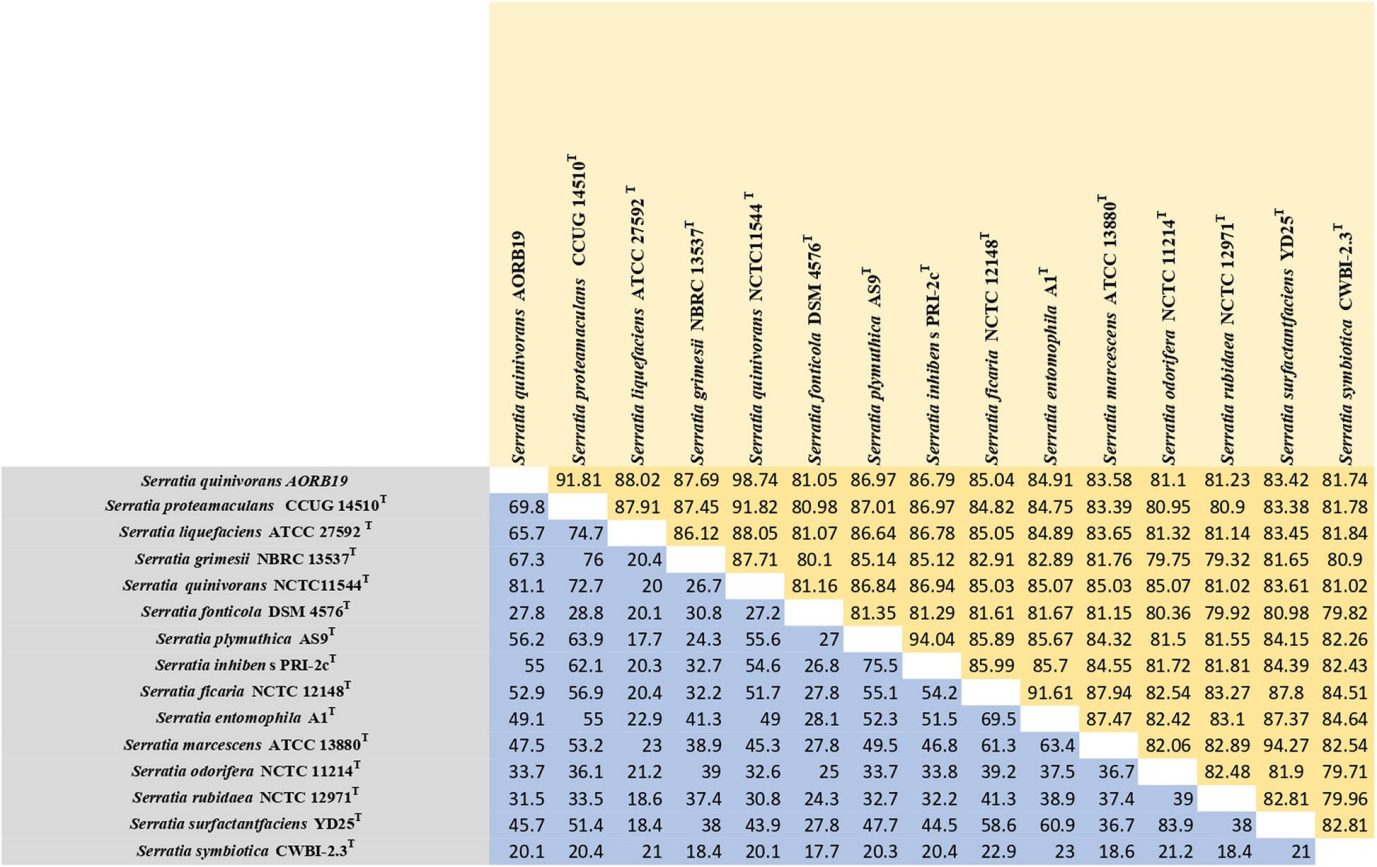
Matrix showing the values of ANI and *in-silico* DDH comparison of S. *quinivorans* AORB19 with 14 other type strains of *Serratia*. The upper half of the matrix (highlighted in yellow) shows the results of ANI analysis whereas the lower half of the matrix (highlighted in blue) shows the results of *in-silico* DDH analysis

ANI is a measure of nucleotide-level genomic similarity between the coding regions of two genomes. The ANI matrix (Fig. 5) reveals that the genome of AORB19 showed an ANI < 95% with most of the other type strains in the genus *Serratia* including the *S. proteamaculans* CCUG 14510. A 95% ANI cutoff is the most frequently used standard for species demarcation [28]. However, AORB19 shows a high ANI value (> 95%) with *S. quinivorans* NCTC11544 and therefore this genome is very close to the *S. quinivorans* genome*.* Genome-to-Genome Distance Calculator (GGDC) analyses among the 15 *Serratia* strains (Fig. 5) yielded *in-silico* DDH estimates greater than 81% between strain AORB19 and *S. quinivorans* NCTC11544 indicating that they potentially belong to the same species. All other 13 pairwise comparisons with AORB19 indicated that they were different species based on 70% species delimitation threshold [29].

**Fig. 6:**
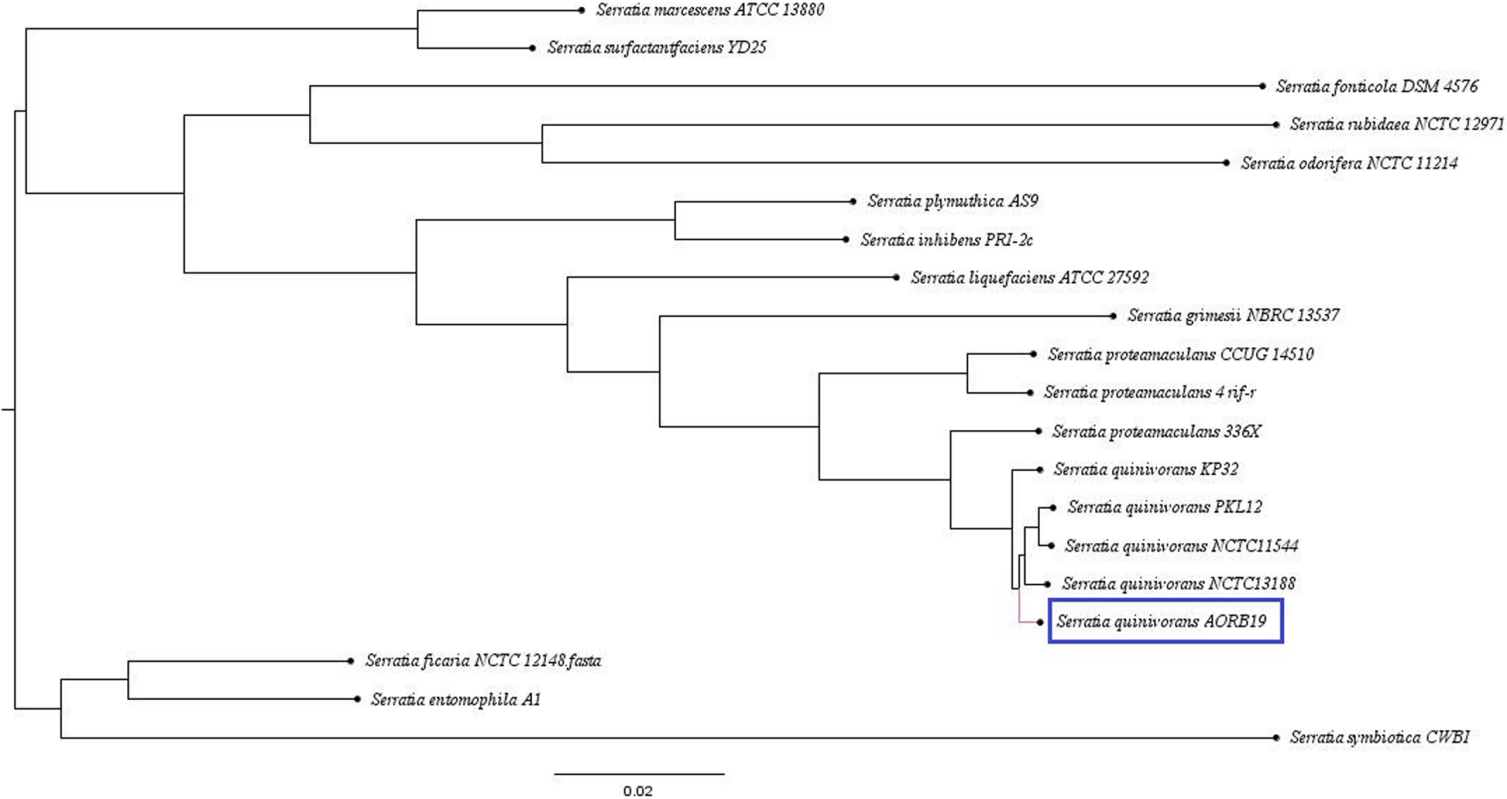
Core genome-based phylogenetic tree constructed by UBCG pipeline

**Fig. 7:**
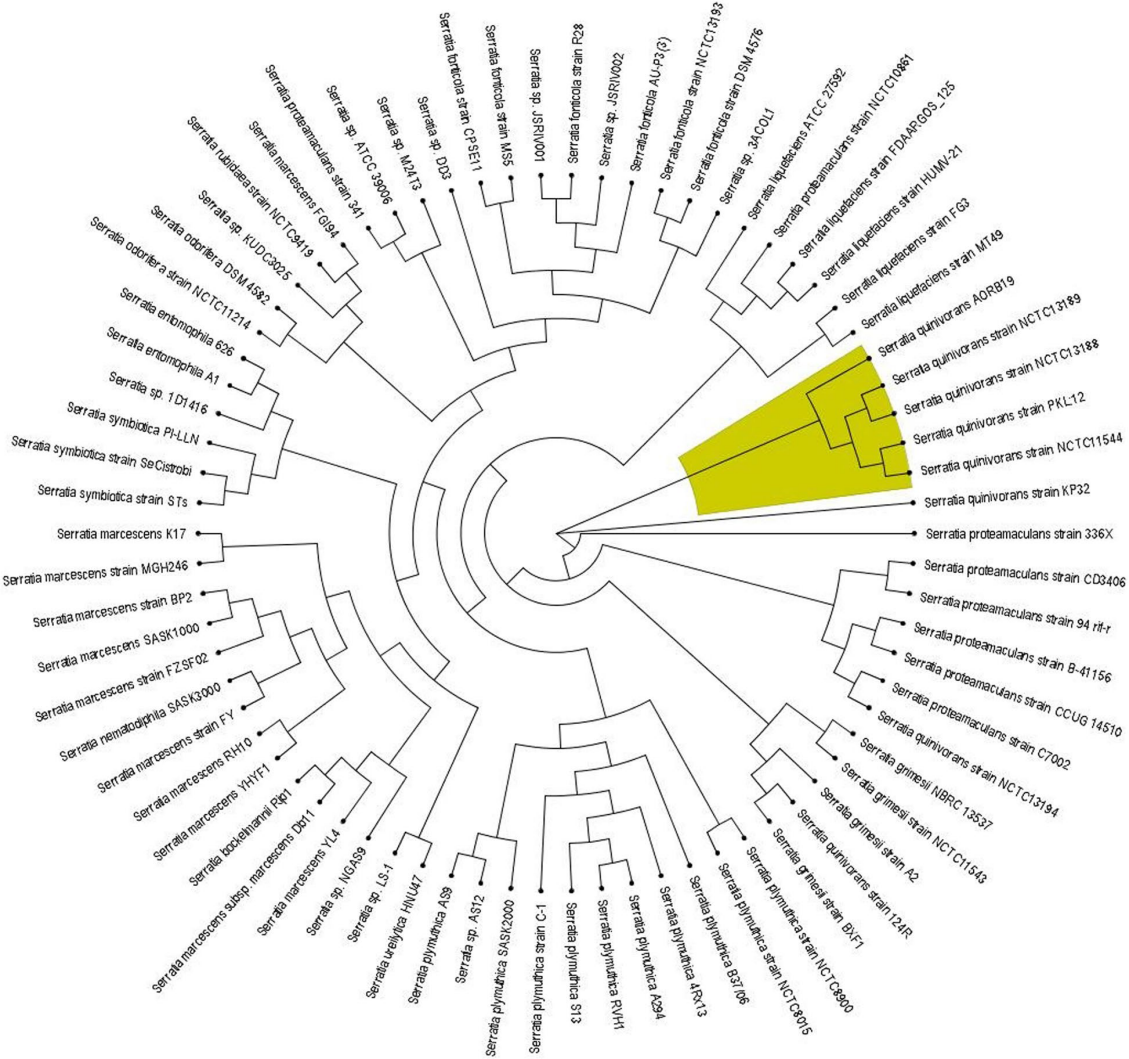
Whole genome phylogeny constructed for *S*. *quinivorans* AORB19 and 74 publicly available *Serratia* genomes. Tree was constructed using the BV-BRC system (https://www.bv-brc.org)

To further infer the phylogenomic relationship of this genome, 16S phylogenetic neighbour joining type tree was constructed. The 16S rRNA tree (Supplementary file 1: Fig. S1) reveals that *S. quinivora* AORB19 is placed in a clade along with *S. proteamaculans, S. liquefaciens, S. grimesii* and *S. quinivorans* CP6a but quite distinctly placed from *S. quinivorans* NCTC 11544 strain. Classical 16S rRNA gene-based trees might not possess sufficient resolution to differentiate between closely related species like *S. liquefaciens, S. grimesii, S. proteamaculans* and *S. quinivorans* [30]. Core genome-based phylogeny provides a better resolution and is more robust than conventional marker gene-based phylogeny. Thus, we investigated the genetic diversity within the *Serratia* genus by inferring the phylogenomic relationship based on the core genome. For this purpose, we used the up-to-date bacterial core gene set (UBCG2) consisting of 81 core genes from 3,508 bacterial species covering 43 phyla [31]. Phylogenetic tree analysis inferred by the maximum-likelihood method based on core genes defined by UBCG2 (Fig. 6) clearly indicated that AORB19 belongs to the same clade as S. *proteamaculans, S. quinivorans, S. liquefaciens* and *S. grimesii* strains. But AORB19 is closer to *S. quinivorans* strains than to *S. proteamaculans* strains in this analysis. The maximum likelihood-based whole genome-based phylogenetic tree (Fig. 7) constructed on 74 publicly available *Serratia* genomes was consistent with the ANI and *in-silico* DDH results. It also corroborated the fact that this genome is closest to the *Serratia quinivorans* species although it had close relationships with *S. proteamaculans* and *S. liquefaciens* strains*.* Despite this strain having been previously reported as *S. proteamaculans* AORB19 [16] based on its 16S rRNA sequence, comparative genomic analyses of the entire genome suggest that the strain should be designated as *Serratia quinivorans* AORB19.

### Lignocellulose degrading enzymes of strain AORB19

CAZyme (carbohydrate-active enzyme) annotation for the AORB19 genome was completed utilizing the dbCAN 3 meta server (https://bcb.unl.edu/dbCAN2/). The different classes of CAZymes, including glycosyltransferase (GT), glycoside hydrolase (GH), polysaccharide lyase (PL), carbohydrate esterase (CE), auxiliary activity (AA), and carbohydrate-binding modules (CBMs), play crucial roles in sugar metabolism, specifically in the synthesis, binding, and breakdown of carbohydrates. The total annotated CAZymes gene number in AORB19 genome was 123 including 47 GTs, 58 GHs, 5 CEs, 8 AAs and 13 CBMs (Supplementary file 2: Fig. S2). No gene was assigned to PLs. A few genes were assigned to more than one class of CAZymes. For example, SQAORB19_5091 was assigned to both CBM32 and GH144 classes.

The glycoside hydrolases in the GH23 family were widely represented in this genome, including 8 predicted encoding genes involved in the deconstruction of peptidoglycan along with chitinase activity. The chitin degrading ability of *Serratia marcescens* has been previously described [32]. The genome of strain AORB19 contains 5 genes encoding GH1, and 5 for GH4s. GH1 members are a widespread group of enzymes that hydrolyze the glycosidic bond between two or more carbohydrate units. The GH1 family comprises the majority of bacterial β-glucosidases used in cellulose hydrolysis [33]. Members of GH4 are also involved in cleavage of glycosidic bonds, but exhibit unusual cofactor requirements for activity involving NAD + [34]. A total of 26 encoded proteins in the AORB19 genome were found to be involved in lignocellulose degradation including chitinase activity (Table 2). These included 10 cellulases, which are key enzymes involved in the decomposition of cellulose into glucose; they are members of the GH1, GH3, GH8, GH43, GH45, GH144 families. A variety of annotated GHs and CEs in AROB19 are predicted to participate in hemicellulose degradation. A total of 4 hemicellulases, including xylanases and mannanases were found in the genome belonging to GH43, CE4 and GH2 families.

**Table 2:** List of potential lignocellulose degrading enzymes found in the genome of strain AORB19

Category	CAZy family	Activities in the family	Gene_id
Cellulase	GH1	β-glucosidase	SQAORB19_2281
			SQAORB19_2395
			SQAORB19_3422
			SQAORB19_1980
			SQAORB19_71
	GH3	β-glucosidase	SQAORB19_518
			SQAORB19_1077
	GH8	polyspecific with cellulase activity	SQAORB19_4140
	GH144	endo-β-1,2-glucanase	SQAORB19_5091
	GH45	Endoglucanase	SQAORB19_591
Xylanase	GH43	β-xylosidase	SQAORB19_2940
	CE4	acetyl xylan esterase	SQAORB19_2919
Mannanase	GH2	β-mannosidase	SQAORB19_418
			SQAORB19_1230
Chitinase	GH18	Chitinase	SQAORB19_4153
			SQAORB19_4595
			SQAORB19_1708
			SQAORB19_3187
	GH23	lysozyme (type G)	SQAORB19_4718
			SQAORB19_2967
			SQAORB19_3012
			SQAORB19_2616
			SQAORB19_3825
			SQAORB19_2092
			SQAORB19_2407
			SQAORB19_270

The second most frequent enzyme family contained in this genome was the glycosyltransferases GT family (47 encoding genes). The transfer of sugar residues from activated donor molecules to saccharide or non-saccharide acceptor molecules to form glycosidic linkages is facilitated by GTs. The most abundant GTs belong to the GT2 family including cellulose synthase, chitin synthase, mannosyltransferase, glucosyltransferase, galactosyltransferase. The output from dbCAN2 also included multiple hits corresponding to carbohydrate esterases (CEs) represented with 5 predicted genes attributed to CE11, CE14, CE4 and CE9 families. CEs accelerate the degradation of polysaccharides by acting on ester bonds in carbohydrates, thereby facilitating the access of glycoside hydrolases. The most abundant CEs in the genome belonged to the CE4 family acting on acetylated xylan and chitin. The genome also had 13 genes predicted to encode carbohydrate-binding modules (CBMs). The majority of predicted CBMs belonged to CBM50 and CBM48. CBM50 proteins play a crucial role in the binding of enzymes with cleavage activity of chitin or peptidoglycan, whereas CBM48 encodes specific modules with glycogen-binding function and is appended to GH13 modules.

The bacterial strain AORB19 also possessed an array of CAZymes and lignin-degrading enzymes (including aromatic compound-degrading and detoxifying enzymes) for the degradation of lignocellulose. CAZymes database (www.cazy.org) includes a class AA (Auxiliary Activities) which hosts a wide range of catalytic modules related to lignocellulose conversion and involved in plant cell wall degradation. The AA class works together with PL, CE and GH enzymes to get access to the carbohydrates within the cell wall of plants and facilitate lignin degradation. Within the AA classes, lignin oxidizing enzymes (LO) are classified into three subclasses including AA1, AA2, and AA3; and lignin degrading enzymes are classified into four: AA4, AA5, AA6, and AA8 [35]. Currently, the AA class encompasses 9 families of ligninolytic enzymes and 7 families of lytic polysaccharide mono-oxygenases. Our study revealed the presence of eight genes encoding ligninolytic enzymes under the AA class in the AORB19 genome. The genes among the AA class were: one in AA1 class which encodes the laccase enzymes, one from AA2 class, two from AA3_2 class, which comprise enzymes like aryl alcohol oxidase and glucose-1-oxidase, and one gene in the AA6 class and three in the AA10 class, known as copper-dependent lytic polysaccharide monooxygenases (LPMOs, Table 3). LPMOs are copper-dependent enzymes with multiple functionalities in plant biomass degradation and play a crucial role in lignin breakdown [36]. The presence of specific genes for different classes of lignin degrading enzymes indicates the strain’s ability for efficient break down of lignin and provides the evidence of the potential of AORB19 to be further genetically modified on specific metabolic pathways and regulatory mechanisms based on practical applications.

**Table 3:** Category and number of annotated open reading frames (ORFs) of auxiliary activities (AA) families in AORB19

Auxiliary Activities Enzymes	Number of ORFs	Annotated Genes IDs	Annotation
AA10	3	SQAORB19_1298, SQAORB19_2413, SQAORB19_3185	Lytic polysaccharide mono-oxygenase, Chitin-binding protein
AA3	2	SQAORB19_928, SQAORB19_1920	Choline dehydrogenase, Glucose-Methanol-Choline (GMC oxidoreductase)
AA6	1	SQAORB19_635	NADPH: quinone oxidoreductase
AA1	1	SQAORB19_2924	multicopper oxidase
AA2	1	SQAORB19_1212	peroxidase/catalase

### Analyses of lignin degradation pathways of *Serratia quinivorans *AORB19

Microbial lignin degradation is a complex process involving multiple enzymes and intermediate products. The specific pathway and products produced may vary depending on the specific microorganisms, conditions involved in the degradation process, as well as source or type of lignin. Degradation process can be broadly categorised into two stages based on conditions and nature of compounds: i) the peripheral, or upper pathway that includes lignin depolymerization to central intermediate products such as catechol and protocatechuate and ii) the central, or lower pathway that includes aromatic ring opening to precursor molecules and compounds such as muconic acid derivatives, acetyl-CoA, succinyl-CoA and pyruvate [35, 37–39].

**Fig. 8:**
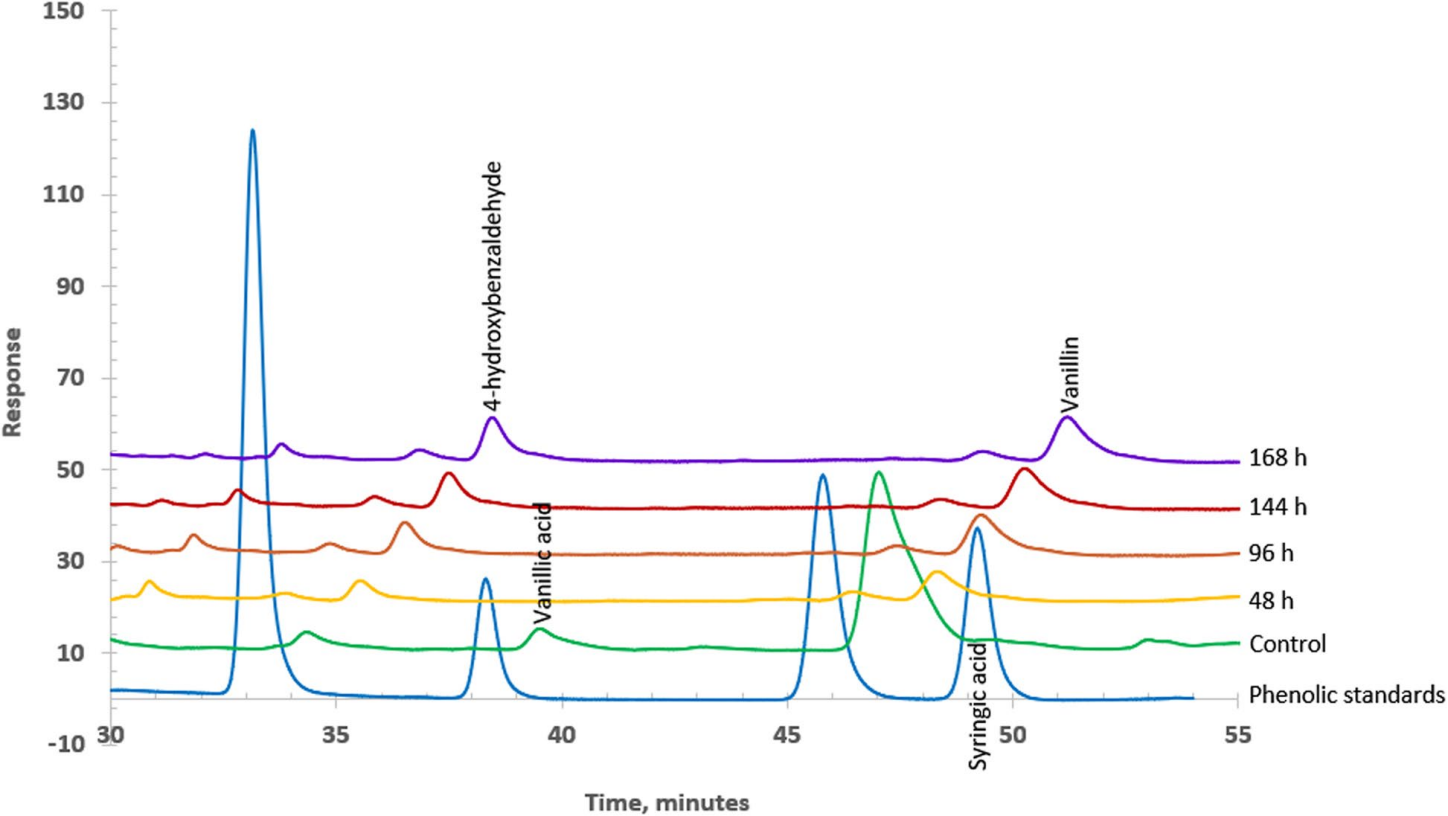
Zoomed LC-UV (λ = 280 nm) chromatograms of Alkali Lignin (Sigma Aldrich) incubated for 168 h without strain (control) or for increasing times (48–168 h) with *Serratia quinivorans* AORB19

Liquid chromatography with UV detection (LC-UV) was employed to identify (using UV spectra) and monitor potential intermediates produced during the degradation of the alkali lignin by *Serratia quinivorans* AORB19 over an 8-d period (Fig. 8; Supplementary file 3: Table S1). Alkali lignin medium without bacterial inoculation was used as a negative control. In the negative control, a number of lignin-derived phenolic compounds were detected including *p*-hydroxybenzaldehyde, vanillic acid, and vanillin. The detection of compounds in the negative control can be attributed to the residual by-products from the alkali treatment during the manufacturing process [40].

In the treated samples, three phenolic compounds were observed with concentrations varying as follows: *p*-hydroxybenzaldehyde, a typical phenolic compound derived from the degradation of lignin, was found to gradually increase over time; vanillin, which experienced a significant decrease after 48 h compared to the control, then gradually rose over the remaining time period of the study; and vanillic acid, which was completely consumed after 48 h, remained undetectable until the end of the experiment (Fig. 8; Supplementary file 3: Table S1). The preponderance of vanillin and vanillic acid in the supernatants is in agreement with the origin of alkali lignin used in this study, *i.e*., softwood. The increasing concentration of* p*-hydroxybenzaldehyde and vanillin in the samples is indicative of lignin degradation by the *Serratia quinivorans* AORB19 bacterium. Both chemicals result from the cleaving oxidation of the benzyl carbon adjacent to the *p-*hydroxyphenyl (H) and guaiacyl (G) units, respectively [41–43]. The *Serratia quinivorans* AORB19 genome harbored several oxidative lignin degrading enzymes including a few potential laccase-like genes—multi multicopper oxidases (SQAORB19_2332; SQAORB19_1606; SQAORB19_2924), DyP-peroxidases (SQAORB19_3208; SQAORB19_3467; SQAORB19_3468) and dehydrogenases (SQAORB19_610; SQAORB19_635) contributing to the formation of the two difunctionalized aldehydes. In similar lines, a thermoalkaliphilic laccase from *Caldalkalibacillus thermarum* TA2.A1 has also been reported to depolymerize kraft lignin to *p*-hydroxybenzaldehyde as a metabolic intermediate [44]. Once formed, the aldehydes can be oxidized into *p*-hydroxybenzoic acid or vanillic acid by the action of dehydrogenase enzymes, as previously reported [45, 46]. In agreement, two aldehyde dehydrogenases genes (SQAORB19_5141; SQAORB19_610) were identified during the genome analyses of strain AORB19. Similarly, an aldehyde dehydrogenase gene responsible for the conversion of syringaldehyde to syringic acid was identified in a previous study [39].

The fact that vanillic acid, present in the controls appeared completely depleted in the presence of *S. quinivorans* AORB19 suggests that the latter has the capability to transform the acid. The genome of strain AORB19 possessed the candidate genes of vanillate O-demethylase oxygenase (SQAORB19_2890; SQAORB19_5632) that is known to convert vanillic acid into protocatechuic acid [47]. The *o*-catechol and protocatechuate obtained from the depolymerization of lignin monomers can then be converted to *cis,cis*-muconate and 3-carboxy-*cis,cis*-muconate, respectively, by the ring opening dioxygenases (SQAORB19_3419; SQAORB19_3420) identified in the genome of strain AORB19. The β-ketoadipate pathway plays a vital role in the degradation of aromatic compounds, including lignin, by enabling microorganisms to utilize carbon from complex polymers, and derives its name from the characteristic intermediate, β-ketoadipate, which serves as a branching point for further metabolism [48–51]. The genome of strain AORB19 also possessed numerous key genes for the β-ketoadipate pathway that could be involved in the degradation of aromatic compounds. SQAORB19_989 and SQAORB19_3417 encode CMD γ -carboxymuconolactone decarboxylase; SQAORB19_1365 encodes ELH, enol lactone hydrolase; SQAORB19_616, SQAORB19_1321, SQAORB19_1366 encode TH, β-ketoadipyl-CoA thiolase; SQAORB19_1367 (subunit B), SQAORB19_1368 (subunit A) encode TR, β-ketoadipate:succinyl-CoA transferase; SQAORB19_3416 encodes CMH, β-carboxymuconolactone hydrolase and CMLE, β-carboxy-cis, cis-muconate lactonizing enzyme; SQAORB19_3419 (alpha chain) and SQAORB19_3420 (beta chain) encode P3,4O, protocatechuate 3,4-dioxygenase.

Notably, genes for additional pathways linked with lignin degradation such as the gentisate, anthranilate, homogentisic and phenylacetate–CoA pathways were also detected in the genome analyses (see Supplementary file 4: Table S2; Supplementary file 5: Table S3). These degradation pathways allow prokaryotes to break down and utilize aromatic compounds as a carbon and energy sources. Taken together, the results of *Serratia quinivorans* AORB19 genome analysis and the formation of detectable traces of transient aromatic compounds in LC-UV analysis validated each other and confirmed the lignin-degradative traits of the strain *Serratia quinivorans* AORB19. More so, with the genetic pathways characterized, the strain *Serratia quinivorans* AORB19 has the potential to undergo genetic modification and optimization in order to enhance the cost-effectiveness and sustainability of lignocellulosic biorefineries, addressing the urgent need for efficient techniques to convert lignin into bioproducts.

### Determination of laccase production using agro-industrial biomasses

**Fig. 9:**
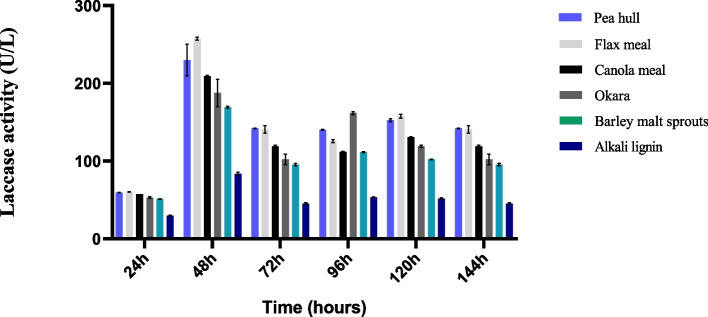
Laccase production by *Serratia quinivorans* AORB19 using different agro-industrial biomasses as carbon source

Considering the strain's inherent capacity for natural extracellular laccase production and its lignin-degradative traits identified through genomic analysis, the strain was tested for its ability to enhance laccase production in various agro-industrial biomasses sourced from Canadian industries. Various industrial waste biomasses including pea hull, flax seed meal, canola meal, okara and barley malt sprouts were used separately as a carbon source to assess their impacts on the strain’s laccase production. It was observed that for all substrates, the enzyme activity maximized at 48 h. At this time, flax seed meal exhibited maximum laccase activity of 257.71 U/L, which was three-fold higher than the laccase activity observed when alkali lignin (Sigma Aldrich) was utilized as the carbon source (83.65 U/L). It was followed by pea hull activity of 230.11 U/L, canola meal activity of 209.56 U/L, okara activity of 187.67 U/L and barley malt sprouts activity of 169.27 U/L (Fig. 9). These results indicated the excellent adaptability of the strain to grow well on a broad range of substrates, particularly on flax seed meal leading to enhanced laccase production. Of note, the strain *Serratia quinivorans* AORB19 efficiently produced laccase enzymes using these heterogeneous biomasses without requiring further substrate optimization.

Microorganisms that encompass broad substrate utilization including lignocellulose-rich plant or agricultural residues are deemed essential for robust enzymatic degradation of lignocellulose [52, 53]. Agro-industrial residues such as pea hull, flax seed meal, canola meal, okara, and barley malt sprouts are the most common feed ingredients for poultry and livestock in Canada [54]. Multiple cohesive enzymes, including laccases are required to facilitate the degradation of intact cell walls of these agro-industrial residues. Apart from the impressive growth characteristics and laccase production observed in this study using flax seed meal, other research has also reported the induction of laccase production in different organisms using various substrates. For instance, pea peels induced laccase production (0.85 U/mL) in *Bacillus aquimaris* AKRC02 through submerged fermentation [55]. Similarly, the fungus *Pleurotus ostreatus* DAOM 197961 was able to produce laccase when grown in canola meal and was found to decrease the meal’s phenolic content upon solid-state fermentation [56]. Moreover, a 2.11-fold increase in laccase production was observed when 0.1% okara was used in submerged fermentation with gram-negative *Rheinheimera sp.* [57].

Bacterial laccases are exceptional environmentally friendly catalysts known for their wide substrate specificity, offering numerous potential applications in areas such as bioremediation, lignocellulose processing, waste valorization and beyond. In the study of bioprocessing using mixed cellulosic feedstocks for ethanol production [58], the increased release of reducing sugars was attributed to the synergistic action of cellulases and ligninolytic laccase, which potentially enhances the accessibility of holocellulolytic enzymes to holocelluloses, consequently leading to improved production of fermentable sugars. Similarly, strain AORB19’s ability to thrive and produce laccase using low-cost raw substrates as carbon sources suggested it may, at the same time, produce holocellulolytic enzymes that synergistically work together with laccase to efficiently hydrolyze lignocellulose. The existence of relevant gene encoding sequences in the genome sequence supports such a hypothesis that can be confirmed by enzymatic activity analyses in future research. Our results suggested the great potential for strain AORB19’s applications in low-cost enzyme production, biomass pretreatment and valorization.

## Conclusion

The complete genome sequence of *Serratia quinivorans* AORB19 strain has unveiled that it harbors a multitude of genes for carbohydrate-active enzymes, which facilitates growth of this bacterium on lignocellulosic biomasses. More so, eight key lignin-degrading enzyme genes in class AA, including lignin oxidizing and lignin degrading genes, were also identified along with an array of enzymes responsible for the degradation of lignin-derived aromatic compounds. The utilization of whole genome sequencing analyses, coupled with LC-UV analyses provided additional evidence supporting the involvement of *Serratia quinivorans* AORB19 in the process of lignin degradation in nature. Furthermore, the demonstrated high potential for laccase production across all tested agro-industrial residues highlights the strain's broad adaptability and underscores its potential application in the valorization of lignocellulosic waste in biorefineries.

The complete genome sequence of *Serratia quinivorans* AORB19 strain has unveiled its extensive repertoire of carbohydrate-active enzyme genes, enabling its proficient utilization of lignocellulosic biomasses. Moreover, the identification of eight crucial lignin-degrading enzyme genes, including those involved in lignin oxidation and degradation within class AA, alongside a diverse set of enzymes responsible for metabolizing lignin-derived aromatic compounds, underscores the strain's pivotal role in lignin breakdown. The integration of whole genome sequencing analyses, along with LC-UV analyses, furnishes additional evidence bolstering the strain's involvement in lignin degradation processes in natural environments. Furthermore, the demonstrated high potential for laccase production across all tested agro-industrial residues highlights the strain's broad adaptability and underscores its potential for application in lignocellulose bioprocessing and valorization of biomass residues within the context of biorefineries.

## Methods

### Chemicals and reagents

The lignin used in this study, *i.e*., Lignin, alkali (CAS8068-05–1, Catalog number 370959), was purchased from Sigma Aldrich (Oakville, ON, Canada). Based on the generic structure provided by Sigma-Aldrich (primarily G units, and an -SH group on the benzylic carbon of the propanoid chain), Lignin, alkali can be assimilated to a partially ionized Kraft lignin obtained from softwood;

The growth medium used in laccase assays was Czapek dox broth containing the following reagents per liter of distilled water: 30.0 g sodium nitrate, 3.0 g magnesium sulfate, 0.5 g potassium chloride, 0.5 g potassium phosphate dibasic, 1.0 g ferrous sulfate, and phenolic substrate 2,6-dimethoxyphenol.

### Microorganism and laccase assays

In this study, a bacterial strain AORB19 isolated using a high throughput screening (HTP) method and characterized from decomposed wood samples [59] was used. The lignin degrading potential of this strain was previously evaluated by its growth in culture media with alkali lignin as the carbon source and laccase activity assays employing quantitative and qualitative methods with different substrates such as 2,2′-azino-bis (3-ethylbenzothiazoline-6-sulfonic acid (ABTS), 2,6-dimethoxyphenol (2,6 DMP), syringaldazine, and guaiacol.

### Genome assembly and annotation

The genomic DNA of strain AORB19 was extracted using DNA isolation kit (Biobasic Inc. Canada) and the next-generation sequencing libraries were sequenced based on the Illumina NovaSeq sequencing platform. FastQC was used to evaluate the quality of the raw reads, and Trimmomatic (v.0.39) [60] was employed to perform trimming of low-quality bases and adapters. Low quality nucleotides and reads that were less than 50 bp were clipped. De novo assembly of genome sequences was performed with SPAdes (v.3.15.4) [61]. The assembled genome contiguity was evaluated with QUAST [62] and genome completeness was assessed with BUSCO [63]. The assembled genome was annotated utilizing RASTk under Patric (3.6.12) (https://www.patricbrc.org). The functional annotation was completed utilizing eggNOG (http://eggnog.embl.de) for COG prediction, whereas, metabolic pathways prediction was completed using KEGG Automatic Annotation Server (KAAS) (https://www.genome.jp/kegg/kaas/). The gene ontology (GO) annotation was carried out using InterProScan 5 [64].

### Whole genome sequence analysis

Average nucleotide identity (ANI) was determined for *Serratia quinivorans* AORB19 genome along with 14 other type strain genomes of *Serratia* using the ANI calculator of EzBiocloud [65]. The ANI values were presented in a matrix-like manner. The ANI cutoff value to circumscribe prokaryotic species boundaries was considered to be approximately 95%. *In-silico* DNA–DNA hybridization (*is*DDH) was also performed using the Genome-to-Genome Distance Calculator (GGDC) (http://ggdc.dsmz.de/home.php) [29]. The estimation of *is*DDH values involves utilizing formula 2 from GGDC, which calculates the sum of identities found in HSPs (High scoring Segment Pairs) divided by the total length of all HSPs [66]. The results of formula 2 were adopted and a cutoff value in DDH was chosen to be 70% for delimitation of species.

### Phylogenetic analysis

16S rRNA phylogeny was constructed using the neighbor-joining algorithm in MEGA11 Software [67]. Bacterial core genome-based phylogeny was also constructed along with 20 other strains of *Serratia* utilizing the UBCG2 pipeline [31]. The UBCG2 pipeline employs the prevailing approach for constructing phylogenetic trees based on core genes. Core genes are defined as: 1) Genes that are present in a majority of species and 2) Genes that are present as a single copy (likely orthologous but not paralogous).

In another approach, a whole genome phylogenetic tree for 74 Serratia genomes was generated using the codon tree method within BV-BRC 3.28.22 (https://www.bv-brc.org) [68]. This method utilizes BV-BRC PGFams as homology groups and analyzes aligned proteins and coding DNA from single-copy genes using the tree building program RAxML [69]. The Codon Tree option was selected with gene number 100. The generated tree was visualized using FigTree v1.4.4 (http://tree.bio.ed.ac.uk/software/figtree/).

### Analysis of CAZymes and mining of lignocellulose degrading genes

The integrated dbCAN3 meta server was used with default settings to classify the carbohydrate active enzyme (CAZyme) encoding genes in the genome of strain AORB19. This classification was performed using the dbCAN database (with HMMER), the CAZy database (with DIAMOND), and the dbCAN-Sub database (with HMMER) [70]. The resulting data was imported into R studio and organized. Manual screening was conducted to further analyze CAZyme genes, and confirmation was based on the requirement that the sequence must be recognized by at least two of the aforementioned databases. The lignocellulose degrading genes were further revealed by cross-checking with the annotations available in CAZy database [71].

### Analytical method for lignin degradation products

The Strain AORB19 was grown in 50 mL of culture medium with 0.1% Lignin, alkali from Sigma Aldrich as the carbon source and cultured at 27 °C on a shaker for 8 d. Every 48 h, a sample was sacrificed and centrifuged at 15,000 rpm for 10 min to remove the bacteria, and the culture supernatant was separated for further analysis. A control sample (culture media devoid of bacterial strain) was also prepared and subjected to the same treatment and analytical path. Phenolic compounds typical of lignin degradation (*i.e*., phenol, *o*-catechol, 3-methylcatechol, guaiacol, syringol, *p*-hydroxybenzaldehyde, *p*-hydroxybenzoic acid, vanillin, vanillic acid, syringaldehyde, syringic acid, acetovanillone, acetosyringone, ferulic acid) were qualitatively monitored in the supernatant using an Agilent 1260 Infinity II HPLC system equipped with a diode array detector (Agilent Technologies, Inc., Santa Clara, CA, USA). Separation of analytes was carried out with a Gemini® NX-C18 column (3 µm, 110 Å, 150 mm × 4.60 mm, Phenomenex, Torrance, CA, USA), at a temperature of 40 °C and a flow rate of 0.5 mL/min. A gradient of 5 to 95% MeOH in 0.044 N H_3_PO_4_ was used and detection was conducted at 280 nm. The injection volume was 100 μL.

### Determination of laccase production using industrial biomasses

Lignocellulosic residues were sourced from various Canadian companies including pea hull (NutriPea), flax seed meal (Pilling Foods), Okara (Sunrise Soya Foods), canola meal (Richardson), and barley malt sprouts (Mad Barn). After being oven dried overnight at 60 °C, the residues were grinded in a coffee grinder and stored at 4 °C. Culture media was prepared using 0.1% biomass waste as carbon source separately, and the strain AORB19 was cultivated using submerged fermentation conditions. The crude enzyme was obtained from the centrifuged culture supernatant subjected to 13,000 rpm for 5 min. The assay mixture, comprising 2,6 DMP (2 mM) in 160 µL of sodium acetate buffer (0.1 M, pH 5), and 50 µL of crude enzyme, added up to a total reaction volume of 210 µL. The change in absorbance, indicative of DMP oxidation, was monitored at 469 nm over a 20-min period at 1-min intervals. Laccase activity was quantified as the enzyme amount required to oxidize 1 µmol of 2,6-DMP within a one-minute time interval, defining one unit of laccase activity [59]. The production of laccase was measured periodically through spectrophotometry and compared to the control media prepared using alkali lignin as the carbon source.

### Statistical analysis

The laccase production by *Serratia quinivorans* AORB19 using different biomass was performed in triplicate, and the results were expressed as mean ± standard error in GraphPad Prism software. Post-hoc comparisons were conducted using GraphPad Prism 9.0.0 by applying one-way ANOVA with Dunnett's multiple tests.

### Supplementary Information

Supplementary Material 1.

Supplementary Material 2.

Supplementary Material 3.

Supplementary Material 4.

Supplementary Material 5.

## Data Availability

All data generated or analyzed during the study are included in this published article and its Supplementary files. The datasets featured in this study are accessible in online repositories. The specific repository or repositories can be located at https://www.ncbi.nlm.nih.gov/, with the accession number (BioProject accession—JAYJMV000000000).

## Abbreviations

AA: Auxiliary activity
AAO: Aryl alcohol oxidases
ANI: Average Nucleotide Identity
BUSCO: Benchmarking Universal Single-Copy Orthologous
BV-BRC: Bacterial and Viral Bioinformatics Resource Center
CBM: Carbohydrate-binding modules
CE: Carbohydrate esterase
DDH: DNA-DNA hybridization
GGDC: Genome-To-Genome Distance Calculator
GH: Glycoside hydrolase
GO: Gene Ontology
GT: Glycosyl transferase
KEGG: Kyoto Encyclopedia of Genes and Genomes
KO: KEGG Orthology
LC-UV: Liquid chromatography with UV detection
LPMO: Lytic polysaccharide monoxygenase
PL: Polysaccharide lyase
UBCG: Up-to-date Bacterial Core Gene
