# Supplementary material for: Uncovering the lignin-degrading potential of Serratia quinivorans AORB19: insights from genomic analyses and alkaline lignin degradation
Source: BMC Microbiol. 2024 May 25;24:181. doi: 10.1186/s12866-024-03331-3 (PMC11127350; doi:10.1186/s12866-024-03331-3)
Supplement: Supplementary file 1 — Supplementary Material 1. [file 12866_2024_3331_MOESM1_ESM.docx]

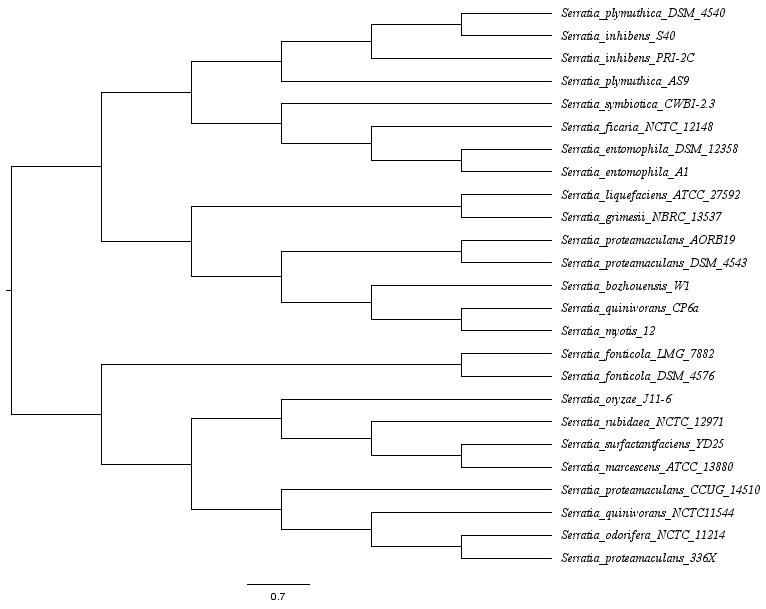


**Figure S1. 16S rRNA phylogenic tree constructed using 25 different strains of Serratia using the NJ algorithm in MEGA Software.**
