# Supplementary material for: Uncovering the lignin-degrading potential of Serratia quinivorans AORB19: insights from genomic analyses and alkaline lignin degradation
Source: BMC Microbiol. 2024 May 25;24:181. doi: 10.1186/s12866-024-03331-3 (PMC11127350; doi:10.1186/s12866-024-03331-3)
Supplement: Supplementary file 3 — Supplementary Material 3. [file 12866_2024_3331_MOESM3_ESM.docx]

| **Table S1.** HPLC-UV qualitative monitoring of phenolic compounds identified in the culture supernatant using Alkali Lignin (Sigma Aldrich) (Control: no strain; time: incubation time with Serratia quinivorans AORB19) | | | | | | | |  |
| --- | --- | --- | --- | --- | --- | --- | --- | --- |
|  |  |  |  |  |  |  |  |  |
| **Phenolic Compounds** | **RT (min)** | **Area (control)** | **Area (48 h)** | **Area (96 h)** | **Area (144 h)** | **Area (168 h)** |  |  |
| 4-Hydroxybenzaldehyde | 33.3 | 186.2022 | 168.2848 | 255.4662 | 279.6373 | 350.1594 |  |  |
| Vanillin | 46.1 | 2756.4584 | 367.5246 | 481.5995 | 478.3785 | 527.6389 |  |  |
| Vanillinic acid | 38.5 | 249.3493 | Nd | nd | nd | nd |  |  |
|  |  |  |  |  |  |  |  |  |
