# Supplementary material for: Uncovering the lignin-degrading potential of Serratia quinivorans AORB19: insights from genomic analyses and alkaline lignin degradation
Source: BMC Microbiol. 2024 May 25;24:181. doi: 10.1186/s12866-024-03331-3 (PMC11127350; doi:10.1186/s12866-024-03331-3)
Supplement: Supplementary file 4 — Supplementary Material 4. [file 12866_2024_3331_MOESM4_ESM.docx]

**Table S2. Main genes responsible for lignin degradation in strain AORB19**
