# Supplementary material for: Uncovering the lignin-degrading potential of Serratia quinivorans AORB19: insights from genomic analyses and alkaline lignin degradation
Source: BMC Microbiol. 2024 May 25;24:181. doi: 10.1186/s12866-024-03331-3 (PMC11127350; doi:10.1186/s12866-024-03331-3)
Supplement: Supplementary file 5 — Supplementary Material 5. [file 12866_2024_3331_MOESM5_ESM.docx]

**Table S3.** Degradative pathways for lignin-derived aromatic compounds

| **Degradative Pathways for Lignin-Derived Aromatic Compounds** | | | | | | |
| --- | --- | --- | --- | --- | --- | --- |
|  | **RAST Subsystem** | **Encoded Protein** | **Gene ID** | **start** | **stop** | **strand** |
| **Peripheral pathways for catabolism of aromatic compounds** | Quinate degradation | 3-dehydroquinate dehydratase II (EC 4.2.1.10) | SQAORB19_313 | 325319 | 325747 | + |
|  |  |  | SQAORB19_2595 | 268065 | 268517 | + |
|  | Benzoate degradation | Benzoate transport protein | SQAORB19_162 | 178218 | 179411 | + |
|  | Biphenyl Degradation | Acetaldehyde dehydrogenase, acetylating, (EC 1.2.1.10) in gene cluster for degradation of phenols, cresols, catechol | SQAORB19_1381 | 197955 | 198842 | + |
|  | Biphenyl Degradation | 4-hydroxy-2-oxovalerate aldolase (EC 4.1.3.39) | SQAORB19_1382 | 198826 | 199827 | + |
|  | Biphenyl Degradation | Acetaldehyde dehydrogenase (EC 1.2.1.10) | SQAORB19_1731 | 557246 | 554574 | - |
|  | Biphenyl Degradation | Biphenyl-2,3-diol 1,2-dioxygenase (EC 1.13.11.39) | SQAORB19_4553 | 16499 | 17008 | + |
|  | p-Hydroxybenzoate degradation | 4-hydroxybenzoate transporter | SQAORB19_2247 | 496432 | 495095 | - |
|  | p-Hydroxybenzoate degradation | P-hydroxybenzoate hydroxylase (EC 1.14.13.2) | SQAORB19_5600 | 2 | 232 | + |
|  |  |  | SQAORB19_2862 | 194919 | 196268 | + |
|  |  |  | SQAORB19_5237 | 3 | 341 | + |
|  |  |  | SQAORB19_3408 | 175141 | 176499 | + |
|  |  |  | SQAORB19_3414 | 180537 | 181892 | + |
|  |  |  | SQAORB19_3415 | 181903 | 183081 | + |
| **Metabolism of central aromatic intermediates** | Catechol branch of beta-ketoadipate pathway | Beta-ketoadipate enol-lactone hydrolase (EC 3.1.1.24) | SQAORB19_1365 | 179426 | 178665 | - |
|  | Catechol branch of beta-ketoadipate pathway | 3-oxoadipate CoA-transferase subunit B (EC 2.8.3.6) | SQAORB19_1367 | 181301 | 180621 | - |
|  | Catechol branch of beta-ketoadipate pathway | 3-oxoadipate CoA-transferase subunit A (EC 2.8.3.6) | SQAORB19_1368 | 181999 | 181313 | - |
|  | Catechol branch of beta-ketoadipate pathway | Succinyl-CoA:3-ketoacid-coenzyme A transferase subunit A (EC 2.8.3.5) | SQAORB19_2005 | 228909 | 229607 | + |
|  | Catechol branch of beta-ketoadipate pathway | Succinyl-CoA:3-ketoacid-coenzyme A transferase subunit B (EC 2.8.3.5) | SQAORB19_2006 | 229618 | 230271 | + |
|  | Salicylate and gentisate catabolism | Maleate cis-trans isomerase (EC 5.2.1.1) | SQAORB19_87 | 98212 | 98964 | + |
|  | Salicylate and gentisate catabolism | Fumarylacetoacetate hydrolase family protein | SQAORB19_93 | 105184 | 106188 | + |
|  | Salicylate and gentisate catabolism | 4-hydroxybenzoate transporter | SQAORB19_2247 | 496432 | 495095 | - |
|  |  |  | SQAORB19_2862 | 194919 | 196268 | + |
|  |  |  | SQAORB19_3408 | 175141 | 176499 | + |
|  |  |  | SQAORB19_3414 | 180537 | 181892 | + |
|  | Salicylate and gentisate catabolism | Gentisate 1,2-dioxygenase (EC 1.13.11.4) | SQAORB19_3657 | 176529 | 175495 | - |
|  | Salicylate and gentisate catabolism | Maleylacetoacetate isomerase (EC 5.2.1.2) | SQAORB19_5358 | 46 | 270 | + |
|  | Protocatechuate branch of beta-ketoadipate pathway | 4-carboxymuconolactone decarboxylase (EC 4.1.1.44) | SQAORB19_989 | 1027933 | 1027616 | - |
|  | Protocatechuate branch of beta-ketoadipate pathway | Beta-ketoadipate enol-lactone hydrolase (EC 3.1.1.24) | SQAORB19_1365 | 179426 | 178665 | - |
|  | Protocatechuate branch of beta-ketoadipate pathway | 3-oxoadipate CoA-transferase subunit B (EC 2.8.3.6) | SQAORB19_1367 | 181301 | 180621 | - |
|  | Protocatechuate branch of beta-ketoadipate pathway | 3-oxoadipate CoA-transferase subunit A (EC 2.8.3.6) | SQAORB19_1368 | 181999 | 181313 | - |
|  | Protocatechuate branch of beta-ketoadipate pathway | Pca regulon regulatory protein PcaR | SQAORB19_1369 | 182179 | 183012 | + |
|  | Protocatechuate branch of beta-ketoadipate pathway | Succinyl-CoA:3-ketoacid-coenzyme A transferase subunit A (EC 2.8.3.5) | SQAORB19_2005 | 228909 | 229607 | + |
|  | Protocatechuate branch of beta-ketoadipate pathway | Succinyl-CoA:3-ketoacid-coenzyme A transferase subunit B (EC 2.8.3.5) | SQAORB19_2006 | 229618 | 230271 | + |
|  |  |  | SQAORB19_3405 | 174043 | 173210 | - |
|  | Protocatechuate branch of beta-ketoadipate pathway | 3-carboxy-cis,cis-muconate cycloisomerase (EC 5.5.1.2) | SQAORB19_3416 | 183215 | 184573 | + |
|  |  |  | SQAORB19_3417 | 184566 | 184955 | + |
|  | Protocatechuate branch of beta-ketoadipate pathway | Protocatechuate 3,4-dioxygenase alpha chain (EC 1.13.11.3) | SQAORB19_3419 | 186610 | 185984 | - |
|  | Protocatechuate branch of beta-ketoadipate pathway | Protocatechuate 3,4-dioxygenase beta chain (EC 1.13.11.3) | SQAORB19_3420 | 187335 | 186607 | - |
|  | 4-Hydroxyphenylacetic acid catabolic pathway | Homoprotocatechuate degradative operon repressor | SQAORB19_2040 | 264357 | 263902 | - |
|  | 4-Hydroxyphenylacetic acid catabolic pathway | 5-carboxymethyl-2-oxo-hex-3- ene-1,7-dioate decarboxylase (EC 4.1.1.68) | SQAORB19_2041 | 264683 | 265315 | + |
|  | 4-Hydroxyphenylacetic acid catabolic pathway | 2-hydroxyhepta-2,4-diene-1,7-dioate isomerase (EC 5.3.3.-) | SQAORB19_2042 | 265312 | 266076 | + |
|  | 4-Hydroxyphenylacetic acid catabolic pathway | 5-carboxymethyl-2-hydroxymuconate semialdehyde dehydrogenase (EC 1.2.1.60) | SQAORB19_2043 | 266073 | 267527 | + |
|  | 4-Hydroxyphenylacetic acid catabolic pathway | 3,4-dihydroxyphenylacetate 2,3-dioxygenase (EC 1.13.11.15) | SQAORB19_2044 | 267537 | 268394 | + |
|  | 4-Hydroxyphenylacetic acid catabolic pathway | 5-carboxymethyl-2-hydroxymuconate delta-isomerase (EC 5.3.3.10) | SQAORB19_2045 | 268404 | 268790 | + |
|  | 4-Hydroxyphenylacetic acid catabolic pathway | 2-oxo-hepta-3-ene-1,7-dioic acid hydratase (EC 4.2.-.-) | SQAORB19_2046 | 268801 | 269604 | + |
|  | 4-Hydroxyphenylacetic acid catabolic pathway | 4-hydroxyphenylacetate symporter, major facilitator superfamily (MFS) | SQAORB19_2048 | 270543 | 271913 | + |
|  | 4-Hydroxyphenylacetic acid catabolic pathway | Transcriptional activator of 4-hydroxyphenylacetate 3-monooxygenase operon, XylS/AraC family | SQAORB19_2049 | 271973 | 272869 | + |
|  | 4-Hydroxyphenylacetic acid catabolic pathway | 4-hydroxyphenylacetate 3-monooxygenase, reductase component (EC 1.6.8.-) | SQAORB19_2051 | 274629 | 275144 | + |
|  | N-heterocyclic aromatic compound degradation | 1H-3-hydroxy-4-oxoquinaldine 2,4-dioxygenase | SQAORB19_1776 | 599632 | 600429 | + |
|  | Central meta-cleavage pathway of aromatic compound degradation | Acetaldehyde dehydrogenase, acetylating, (EC 1.2.1.10) in gene cluster for degradation of phenols, cresols, catechol | SQAORB19_1381 | 197955 | 198842 | + |
|  | Central meta-cleavage pathway of aromatic compound degradation | 5-carboxymethyl-2-hydroxymuconate semialdehyde dehydrogenase (EC 1.2.1.60) | SQAORB19_2043 | 266073 | 267527 | + |
|  | Central meta-cleavage pathway of aromatic compound degradation | 3,4-dihydroxyphenylacetate 2,3-dioxygenase (EC 1.13.11.15) | SQAORB19_2044 | 267537 | 268394 | + |
|  | Central meta-cleavage pathway of aromatic compound degradation | 5-carboxymethyl-2-hydroxymuconate delta-isomerase (EC 5.3.3.10) | SQAORB19_2045 | 268404 | 268790 | + |
|  | Central meta-cleavage pathway of aromatic compound degradation | 2-oxo-hepta-3-ene-1,7-dioic acid hydratase (EC 4.2.-.-) | SQAORB19_2046 | 268801 | 269604 | + |
|  | Central meta-cleavage pathway of aromatic compound degradation | Protocatechuate 4,5-dioxygenase beta chain (EC 1.13.11.8) | SQAORB19_5222 | 438 | 1 | - |
| **Metabolism of Aromatic Compounds - no subcategory** | Aromatic Amin Catabolism | 3,4-dihydroxyphenylacetate 2,3-dioxygenase (EC 1.13.11.15) | SQAORB19_2044 | 267537 | 268394 | + |
|  | Aromatic Amin Catabolism | 4-hydroxyphenylacetate 3-monooxygenase, reductase component (EC 1.6.8.-) | SQAORB19_2051 | 274629 | 275144 | + |
|  | Aromatic Amin Catabolism | Phenylacetaldehyde dehydrogenase (EC 1.2.1.39) | SQAORB19_4047 | 247169 | 248668 | + |
|  | Gentisate degradation | Fumarylacetoacetate hydrolase family protein | SQAORB19_93 | 105184 | 106188 | + |
|  | Gentisate degradation | 4-hydroxybenzoate transporter | SQAORB19_2247 | 496432 | 495095 | - |
|  |  |  | SQAORB19_2862 | 194919 | 196268 | + |
|  |  |  | SQAORB19_3408 | 175141 | 176499 | + |
|  |  |  | SQAORB19_3414 | 180537 | 181892 | + |
|  | Gentisate degradation | Gentisate 1,2-dioxygenase (EC 1.13.11.4) | SQAORB19_3657 | 176529 | 175495 | - |
|  | Gentisate degradation | Maleylacetoacetate isomerase (EC 5.2.1.2) | SQAORB19_5358 | 46 | 270 | + |
| **Other Peripheral Pathways** | anthranilate pathway | Indole-3-glycerol phosphate synthase (EC 4.1.1.48) / Phosphoribosylanthranilate isomerase (EC 5.3.1.24) | SQAORB19_1769 | 593917 | 592556 | - |
|  | anthranilate pathway | Anthranilate synthase, aminase component (EC 4.1.3.27) | SQAORB19_1772 | 597078 | 595516 | - |
|  | anthranilate pathway | Para-aminobenzoate synthase, amidotransferase component (EC 2.6.1.85) | SQAORB19_3698 | 220497 | 221072 | + |
|  | anthranilate pathway | Anthranilate phosphoribosyltransferase (EC 2.4.2.18) | SQAORB19_1770 | 594919 | 593921 | - |
|  | anthranilate pathway | Anthranilate synthase, amidotransferase component (EC 4.1.3.27) | SQAORB19_1771 | 595516 | 594935 | - |
|  | anthranilate pathway | Aromatic-amino-acid aminotransferase (EC 2.6.1.57) | SQAORB19_1314 | 130448 | 131635 | + |
|  | anthranilate pathway | Tryptophan 2,3-dioxygenase (EC 1.13.11.11) | SQAORB19_1386 | 201487 | 202329 | + |
|  | homogentisic pathway | Phenylalanyl-tRNA synthetase beta chain (EC 6.1.1.20) | SQAORB19_254 | 270624 | 268237 | - |
|  | homogentisic pathway | Phenylalanyl-tRNA synthetase alpha chain (EC 6.1.1.20) | SQAORB19_255 | 271622 | 270639 | - |
|  | homogentisic pathway | ShikiF5-dehydrogenase I alpha (EC 1.1.1.25) | SQAORB19_3776 | 277216 | 278034 | + |
|  | phenylacetate-CoA pathway | Phenylacetate-coenzyme A ligase (EC 6.2.1.30) | SQAORB19_1320 | 138605 | 137295 | - |
|  | phenylacetate-CoA pathway | 3-hydroxyadipyl-CoA dehydrogenase | SQAORB19_1323 | 141779 | 140259 | - |
|  | phenylacetate-CoA pathway | 1,2-epoxyphenylacetyl-CoA isomerase (EC 5.3.3.18) | SQAORB19_1324 | 142573 | 141782 | - |
|  | phenylacetate-CoA pathway | 2,3-dehydroadipyl-CoA hydratase (EC 4.2.1.17) | SQAORB19_1325 | 143350 | 142577 | - |
|  | phenylacetate-CoA pathway | 1,2-phenylacetyl-CoA epoxidase, subunit E (EC 1.14.13.149) | SQAORB19_1326 | 144418 | 143360 | - |
|  | phenylacetate-CoA pathway | 1,2-phenylacetyl-CoA epoxidase, subunit D (EC 1.14.13.149) | SQAORB19_1327 | 144924 | 144427 | - |
|  | phenylacetate-CoA pathway | 1,2-phenylacetyl-CoA epoxidase, subunit C (EC 1.14.13.149) | SQAORB19_1328 | 145695 | 144934 | - |
|  | phenylacetate-CoA pathway | 1,2-phenylacetyl-CoA epoxidase, subunit B (EC 1.14.13.149) | SQAORB19_1329 | 145991 | 145704 | - |
|  | phenylacetate-CoA pathway | 1,2-phenylacetyl-CoA epoxidase, subunit A (EC 1.14.13.149) | SQAORB19_1330 | 146940 | 146002 | - |
|  | resorcinol pathway | 4-hydroxythreonine-4-phosphate dehydrogenase (EC 1.1.1.262) | SQAORB19_2147 | 376509 | 375517 | - |
|  | resorcinol pathway | 2-polyprenyl-6-methoxyphenol hydroxylase | SQAORB19_2838 | 172475 | 171297 | - |
